# Enhancement of Chloroprene/Natural/Butadiene Rubber Nanocomposite Properties Using Organoclays and Their Combination with Carbon Black as Fillers

**DOI:** 10.3390/polym13071085

**Published:** 2021-03-29

**Authors:** Patricia Castaño-Rivera, Isabel Calle-Holguín, Johanna Castaño, Gustavo Cabrera-Barjas, Karen Galvez-Garrido, Eduardo Troncoso-Ortega

**Affiliations:** 1 Unidad de Desarrollo Tecnológico, Universidad de Concepción, Avda. Cordillera 3624, Coronel 4191996, Chile; i.calle@udt.cl (I.C.-H.); g.cabrera@udt.cl (G.C.-B.); k.galvez@udt.cl (K.G.-G.); 2 Facultad de Ingeniería y Tecnología, Universidad San Sebastián, Lientur 1457, Concepción 4080871, Chile; johanna.castano@uss.cl; 3 Laboratorio de Recursos Renovables, Centro de Biotecnología, Universidad de Concepción, Barrio Universitario S/N, Concepción 4070386, Chile; etroncoso@udec.cl

**Keywords:** chloroprene rubber composite, nanocomposites, organoclays, carbon black, mechanical properties, characterization

## Abstract

Organoclay nanoparticles (Cloisite^®^ C10A, Cloisite^®^ C15) and their combination with carbon black (N330) were studied as fillers in chloroprene/natural/butadiene rubber blends to prepare nanocomposites. The effect of filler type and load on the physical mechanical properties of nanocomposites was determined and correlated with its structure, compatibility and cure properties using Fourier Transformed Infrared (FT-IR), X-ray Diffraction (XRD), Thermogravimetric Analysis (TGA) and rheometric analysis. Physical mechanical properties were improved by organoclays at 5–7 phr. Nanocomposites with organoclays exhibited a remarkable increase up to 46% in abrasion resistance. The improvement in properties was attributed to good organoclay dispersion in the rubber matrix and to the compatibility between them and the chloroprene rubber. Carbon black at a 40 phr load was not the optimal concentration to interact with organoclays. The present study confirmed that organoclays can be a reinforcing filler for high performance applications in rubber nanocomposites.

## 1. Introduction

In the plastic and rubber industry, fillers are additives widely used to improve the physical chemical and mechanical properties of materials and their blends. Over the last decade, several efforts have been made to replace conventional reinforcing fillers (e.g., carbon black, CaCO_3,_ and silica) used in bulk amounts during elastomer production with a lower amount of nanofillers [1]. Nanofillers have emerged as a promising alternative to mitigate the pollution caused by the usual rubber fillers [2], which is why several studies have focused on this matter [3,4]. Nanoclays have attracted considerable attention as the most commonly used nanostructured fillers in rubber compounding. Due to their small particle size and large surface area, it is possible to obtain materials with better properties than macro fillers. Moreover, a lower amount of nanofillers has a significant impact on the final nanocomposite properties [5,6]. It is a fact that the size of the filler directly affects the physical and mechanical properties of the nanocomposites [7], as well as the type of structure, amount of filler, and modification of the nanoparticle surface. It has already been reported that blends are capable of providing synergy in the final properties of the product that cannot be achieved individually by any of the components, which constitutes an important way to generate new and differentiating materials [8]. For example, blends of natural rubber (NR) with styrene butadiene rubber (SBR) and nitrile butadiene rubber (NBR) are significant due to a combination of properties, such as good abrasion resistance and excellent oil resistance [9], while those with chloroprene rubber (CR) are noticed for their good weather resistance [10]. There are previous works on elastomeric nanocomposite preparation [7,11,12]; they were based on natural, nitrile and silicon rubber, styrene butadiene rubber (SBR) and epoxidized natural rubber (ENR). However, nanocomposite rubber made from chloroprene and its blends has scarcely been researched.

Chloroprene rubber (CR) is well known for its high rubber strength caused by strain-induced crystallization. However, CR shows deficiencies in its resistance to abrasion [13]. Due to the halogen group in the rubber structure, it resists burning inherently better than exclusively hydrocarbon rubbers [14]. The CR/NR/BR (chloroprene rubber, natural rubber, and butadiene rubber) blend can be used for engineering applications in which high performance, excellent tribological properties, and chemical resistance are required (e.g., mining and bearing industry). Nevertheless, a short operating life-time due to higher abrasion is a common problem in mineral manufacturing. The development of optimal wear resistant materials is based on the design of a tribological system based on the mechanical, chemical, and thermal demands of the environment to which the material will be exposed. Hence, the interest of the mining and mineral processing industries in using wear resistant materials adapted or developed by multidisciplinary groups [15].

The most used strategy to improve the wear phenomenon, from the point of view of the development of new materials, is the study of nanostructure reinforcement. Nanoclays, carbon nanotubes, and graphene are currently very studied as reinforcing additives in elastomeric materials [16,17,18]. The most critical factors in improving rubber composite properties by nanostructures are nanofiller distribution and compatibility with the rubber matrix. For example, the nanocomposite based on graphene oxide (GO)-supported zinc oxide hybrids rubber, prepared though electrostatic adsorption and in situ growth method, exhibits an improvement in the mechanical properties of its crosslinking network [19]. Lingmin et al. (2020) [20] described a new green strategy to increase the mechanical properties of chloroprene rubber (CR). In this study, carbon nanodots (N-CDs) were used as a crosslinker and reinforcing nanofiller. The resulting nanocomposite showed good N-CDs dispersion in the CR matrix without aggregates. A recent report used the gel compounding (GC) method to prepare nitrile-butadiene rubber (NBR)/nanoclay nanocomposites by compounding solid rubber with nanoclay gels [21]. The increase in mechanical properties (250%) was attributed to the uniform and higher dispersion of the nanoclays into the NBR matrix as exfoliated platelets.

This work investigated the use of organoclays fillers, with and without carbon black, on the physical mechanical properties of CR/NR/BR blends required for high-performance applications, specifically those with higher abrasion resistance. For this purpose, CR/NR/BR blend composites were prepared using different concentrations of two Cloisite^®^ organoclays. The same composite formulations, including a fixed amount of carbon black, were also prepared. The resulting composites were characterized according to their physical mechanical, thermal, rheological and structural properties. 

## 2. Materials and Methods

### 2.1. Materials

Chloroprene rubber (CR) of the TW type (Dupont^TM^, Wilmington, DE, USA) stands out for having a high thermal stability during storage, a required organic acceleration and for being suitable for the extrusion and calendaring processes. Natural rubber (NR) of the SGR-10 type (Mavalle S.A.S, Villavicencio, Colombia), butadiene rubber (BR) (Budene 1208, Goodyear Tire & Rubber Company, Akron, OH, USA) and the CR were used for the composite matrices of this work. The Mooney viscosity of the rubbers was of 42–52 (ML 1 + 4, 100 °C) for CR, 75.7 for the NR and 46 for BR, according to the datasheets. Zinc oxide (ZnO, 99.5%), unsolvable sulfur (S), and other additives were used as received and purchased from a local manufacturing company (Rubber Mix S.A.) in Santiago, Chile. The other additives were phenylenediamine antioxidants, a sulfenamide accelerator, stearic acid or stearin, a PVI retardant of the phthalimide type, magnesium oxide and fatty acid process aids. The carbon black (CB) used in this work is of the type N330 (Continex^TM^, Houston, TX, USA), with a particle size of 29 nm. Organoclays, Cloisite^®^ 10A (C10A) and Cloisite^®^ 15 (C15) of the montmorillonite type were purchased from BYK (BYK Additives Co. Ltda., Shangai, China), with a general formula of (Ca,Na,H)(Al,Mg,Fe,Zn)_2_ (Si,Al)_4_O_10_(OH)_2_XH_2_O and a structure made up of silicate layers, sandwiching an aluminum oxide/hydroxide layer (Al_2_(OH)_4_) [22]. The chemical difference among them is the organic modifier in their intergallery spacing between their stacked layers, denoted as d_001_ which is specified in Table 1. 

### 2.2. Compounding and Vulcanization of Samples

The nanocomposites were prepared on an open two roll mill (Lab Tech LRM-M-100, Labtech Engineering Co., Ltd., Mueang Samut Prakan, Thailand), with a friction ratio of 1:1.2 at room temperature. The compounding process started with the mastication of the rubbers in order to reduce their molar mass, aided by an aromatic hydrocarbon resin (ultra-blend^TM^ 5000, Performance Additives, New Philadelphia, OH, USA) that acted as homogenizer of the CR, NR and BR rubbers. After that, the auxiliary additives (i.e., phenylenediamine antioxidants, stearin, magnesium oxide and fatty acid process aids) were added. At this state, blending was left to rest for 45 min to prevent it from heating. Afterward, the additives that affect the start and rate of the vulcanization reaction were added and mixed (i.e., ZnO, S, sulfenamide accelerator and PVI retardant). Nanocomposites with organoclays were prepared by adding the clay directly to CR during its mastication because it is more compatible with this rubber than with NR or BR [23]. Nanoclays were dried in an oven at 100 °C for 24 h before their incorporation into the composites in order to remove lingering water. For composites prepared with CB, the filler was added after the auxiliaries and before the rest stage. All rubber composites had a conventional sulfur vulcanization system. This means that the concentration ratio of accelerants/sulfur was about 0.4. Therefore, the prevailing cross-linking are of the poly-sulfidic type, and this has consequences on some properties, such as a high compression set and a low resistance to reversion (thermal degradation after full cure) [24]. The recipes for the nanocomposites can be seen in Table 2, in which others included phenylenediamine antioxidants (1.1 phr), sulfenamide accelerator (0.9 phr), stearic acid or stearin (1.0 phr), PVI retardant of the phthalimide type (0.5 phr), magnesium oxide (0.8 phr) and fatty acid process aids (2.5 phr). After compounding, composites were vulcanized in an electrically heated hydraulic press (Zhongli Instrument Technology Co, Ltd.; Dongguan, China) at 160 °C to obtain sheets and cylinders of 1.6 and 10 mm in thickness, respectively, according to their cure characteristics (Section 2.3.5) and to obtain the characterization samples.

### 2.3. Characterization Techniques

#### 2.3.1. Physicomechanical Properties

Tensile and tear tests were performed using a universal testing machine (SmarTens 005, Karg Industrietechnik, Krailling, Germany) with a load cell of 5 kN, at a crosshead speed of 500 mm/min and room temperature and following ASTM D412 and ASTM D624 standards. Dumbbell and angle trouser specimens, type C, were used for tension and tear tests, respectively. These specimens were obtained by punching the sheets of the cured nanocomposites with suitable dies in such a way that the longitudinal axis of the specimens was parallel to the composites flow direction in rolling. Hardness was measured on specimens with a 10 mm thickness according to ASTM D 2240 standard with a durometer (Enpaix, Polygon Instrument Limited, Shenzhen, China). Abrasion tests were performed according to ASTM D 5963 standard (A-method) without rotation of the specimen and on a roller abrader machine (Gibrite, Bergamo, Italy). Density was measured in an analytical balance with density kit (Radwag, Radom, Poland), following ASTM D 792 standards. Five specimens were tested for these properties.

#### 2.3.2. X-Ray Diffraction Analysis

X-ray diffraction (XRD) was used to study the solid estate structure of organoclays and its dispersion in the cured composites. The measurements were carried out in a Rigaku Geigerflex diffractometer (Rigaku Corp., Tokyo, Japan), with a Fe Kα radiation of 1.937355 Å (35 kV, 15 mA). The diffraction patterns were recorded from 2θ: 1.5 to 2θ:10° with a step of 0.02°/s. The measurement time for the intensity at each angle was 14 s. The intergallery spacing of the organoclay (d_001_) was derived from the peak position (001) in the X-ray patterns, following Bragg’s law (d = λ/2sin θ).

#### 2.3.3. Thermogravimetric Analysis (TGA) 

The degradation temperature of the cured composites was studied using a thermogravimetric analyzer (TGA) (NETZSCH 209 F3, Selb, Germany). Samples were analyzed from 25 to 600 °C, at a heating rate of 10 °C/min under N_2_ atmosphere.

#### 2.3.4. Fourier Transformed Infrared (FT-IR) Analysis

Sheets around 1.6 mm thick of the cured composites were used as samples for the FT-IR analyses. FT-IR spectra were recorded at 25 °C in a spectrometer (Jasco Inc., Easton, MD, USA) using the Attenuated Total Reflection (ATR) mode in the range of 4000–400 cm^−1^ with a resolution of 4 cm^−1^ and after 64 scans.

#### 2.3.5. Cure Characteristics

The cure characteristics of rubber composites were determined at 160 °C in a moving die rheometer (MDR 2000, MonTech^®^ Rubber Testing Solutions, Buchen, Germany). Scorch time, or initiation of the vulcanization reaction (ts_2_); optimum cure time, or time to 90% cure state (t_90_); minimum torque (M_L_) and maximum torque (M_H_) were evaluated.

## 3. Results and Discussion

### 3.1. Physicomechanical Properties

#### 3.1.1. Tear Properties

The mechanical properties results, as seen in Table 3, showed that organoclays increased the tear strength of the CR/NR/BR nanocomposites by increasing their content up to 7 phr, from which this property seems to remain constant. Thus, the tear strength of A10-7 nanocomposite was of 31.51 N/mm in comparison to the A0 composite (CR/NR/BR rubber matrix without fillers), which was of 16.29 N/mm (93% increase). The same effect was observed for A15-7 nanocomposite with a tear strength of 28.82 N/mm (77% increase, compared to A0). These results were similar to those reported by other authors at 5 phr of Cloisite^®^ 15 in a single CR rubber matrix and with a tear strength of 27.1 N/mm [25]. The carbon black (CB) N330 at 40 phr improved tear strength, acting alone, on the CR/NR/BR rubber matrix. Thereby, the tear strength of B0 nanocomposite was of 27.97 N/mm, which represented a 72% increase on it when compared to the A0 composite. However, the interaction between organoclays and CB did not improve tear strength in any of the organoclay concentrations. This result suggests that 40 phr is not the optimal amount of CB required to obtain a good interaction with C10A or C15 organoclays in the CR/NR/BR rubber matrix.

#### 3.1.2. Tensile Properties

Results of the tensile properties appear in Table 3, where it can be seen that the tensile strength was determined as the maximum load value of the stress–strain curve, while M300 was the tensile stress (or tensile modulus) at 300% elongation. It was observed that the tensile strength of CR/NR/BR nanocomposites was improved by the effect of organoclays. Thereby, the maximum tensile strength value was of 13.65 MPa in the A10-7 nanocomposite. If standard deviations of tensile strength values are considered, it could be observed that the A15-5 and A15-7 nanocomposites had the similar tensile strength values (13.42 ± 0.83 MPa and 12.40 ± 0.85 MPa, respectively) as the A10-7 nanocomposite (13.65 ± 0.76 MPa). Thus, organoclay amounts higher than 5 phr in nanocomposites made the tensile strength remain constant or reduced it. This could be attributed to the partial agglomeration of the organoclay above this limiting concentration [26]. Moreover, nanocomposites with the CB filler at 40 phr did not show higher tensile strength values than those prepared only with organoclays (e.g., A10-7, A15-5, A15-7). These results showed a better interaction in organoclays with rubber phases than in those with the conventional filler of carbon black because the enhancement of the tensile strength of nanocomposites reflected the efficiency in stress transfer from the rubber phases to fillers [27].

On the other hand, the carbon black and organoclays fillers used in the composites of this work showed a lack of synergy between them, which was more evident among CB and C10A, than CB and C15. For example, the nanocomposite B10-7 showed just an 8% increase in its tensile strength compared with the B0 nanocomposite, while the nanocomposite B15-7 showed a 23% increase in this property in contrast to the B0 nanocomposite. This implies that the slight difference in the interaction of CB with C15 organoclay at 7 phr than this of CB with C10A could be due to the higher intergallery spacing of C15.

The composite with the organoclay C15 at 5 phr and carbon black presented a result that did not follow the trend. Thus, the B15-5 composite presented a lower tensile strength (7.02 ± 0.63 MPa) than B15-3 (12.77 ± 0.71 MPa) and B15-7 (12.43 ± 0.40 MPa), but also equal to B15-10 (7.31 ± 0.02 MPa). It was expected that at a 5 and 7 phr load of organoclay with carbon black in the composites, the tensile strength improved until a maximum or similar value, which was not observed at 5 phr. It was also expected that at higher contents than 7 phr, this property would decline, which indeed was observed. This could be attributed to the difficulty in obtaining a compounding with an effective dispersion of the organoclays and carbon black, when high contents of the carbon black as 40 phr were used.

The tensile modulus (M 300) is a property that is related to the degree of stiffness of a composite. In the composites using only organoclay as filler, the highest M 300 value was obtained in the A10-7 composite in which the modulus increased by 126%, in contrast to the A0 composite. The addition of CB to the composites at 40 phr dramatically increased the modulus values by 500%, indicating an increase in matrix rigidity. Regarding the effect of organoclays in combination with CB filler, the highest modulus values were observed in B15-3 (7% increase) and B10-7 (15% increase) composites, followed by B15-3 (7% increase) and B15-10 (5% increase), respectively. An increment in rubber composites rigidity was achieved in all those cases.

Regarding the elongation at break, composites using only organoclays as fillers showed equal or higher values of this property than those with the A0 composite. For instance, an enhancement of about 13% was reached in the A10-5 and A10-7 composites, in contrast to those with the A0 composite, whereas a 25% increase was obtained in composites with A15-5 and A15-7. Those with carbon black did not show the same behavior, because their elongation at break values were always lower than those of the A0 composite. This result proved very positive for organoclays as fillers, because apart from showing a reinforcing effect in rubber composites, it did not impair their flexibility as usually happens with conventional fillers, such as silica and carbon black.

In previous research, it was found that there is a synergistic effect between nanoclays and carbon black as fillers in particular quantities of polymer composites. For example, an optimal combination was at 5 and 15 phr of a montmorillonite organoclay and carbon black of the type N550, respectively [25]. Other researchers [28] showed that by using 10 phr of organoclay bearing octadecylamine and 20 phr of carbon black (N330) in SBR nanocomposites, the tensile strength, elongation at break and stress at 100% elongation increased by a synergistic effect. Etika et al. [29] reported that epoxy composites containing a ratio of 1:2 (wt/wt) between carbon black (42 nm of particle size) and a natural montmorillonite clay provided significant improvement in the storage modulus and electrical conductivity of epoxy composites, in contrast to these same composites with carbon black and without clay.

These previous works reaffirmed our assumption that in our work, the carbon black N330 at 40 phr and with 3, 5, 7 or 10 phr of the C10A and C15 organoclays did not have a good enough interaction between them to improve the tensile and tear properties of CR/NR/BR nanocomposites.

#### 3.1.3. Abrasion Resistance

The abrasion resistance of CR/NR/BR nanocomposites was determined in terms of volume loss, following the ASTM D 5963 standard. The abrasion of rubber is complex and involves more than one mechanism [30]. The results in Table 4 showed that the volume loss by abrasion of composites with organoclay and CB was lower than that with the A0 composite. This reduction depended on the concentration and the type of filler used. All composites prepared with organoclay exhibited a remarkable increase in abrasion resistance in the range between 40 and 46%, and the A10-5 and A15-5 composites showed the highest ones. However, the A15-10 composite increased the least by 11%. This indicated that above a concentration of 7 phr of organoclays, the distribution and dispersion of clay nanoparticles are not homogeneous and could be generating agglomerations that do not allow the reinforcement of abrasion resistance. An improvement of 6.57% in the abrasion resistance of rubber nanocomposites was reported by other authors [31]. They used an organically modified nanoclay (kaolin at 5 phr) with precipitated silica (25 phr) for the hybrid reinforcement of a natural rubber prepared by a roll mixing mill. Meanwhile, Sreelekshmi et al. (2017) [32] reported a 19% increase in abrasion resistance on natural rubber mixed with modified kaolin at 6 phr. From the results, we can deduce that the nanoclay addition of 5 phr is enough to reach a considerable abrasion resistance in these rubber nanocomposites.

The addition of CB at 40 phr into the CR/NR/BR matrix (B0 composite) produced an increase in abrasion resistance of up to 36% in contrast to that of A0. Furthermore, the composites reinforced with organoclay and those combined with CB exhibited an increase in abrasion resistance more similar to that of the A0 composite (30–37%) than those obtained with only organoclays. Moreover, composites prepared with the C15 organoclay and combined with CB showed a higher decrease in volume loss values than those obtained with C10A. These results can be due to the better dispersion and distribution of C15 in the rubber matrix than those of C10A, because of its higher d_001_, as shown by X-ray results (see Section 3.2).

Again, the addition of 10 phr nanoclays to the composite produces the lowest effect on the rubber matrix. Other authors reported similar results when modifying different types of nanoclays with tetrafluoride to include it into natural rubber [33]. They proposed that the improvement in mechanical properties is a consequence of particle dispersion, adequate compatibility among the components, small size and large surface area.

#### 3.1.4. Hardness and Density

Hardness is defined as the mean pressure used to cause materials to undergo plastic deformation. As shown in Table 4, the nanocomposites’ hardness depends on the type of organoclay and added concentration. The B0 composite showed a higher hardness value than the A0 composite, corroborating the use of carbon black as a conventional reinforcement of synthetic rubbers [15]. In all cases, incorporating organoclay and CB produced an increase in hardness values compared to the value shown in B0 and A0. The largest increase in hardness was observed in the B15-5 composite (66 shore A). These results support those reported by Lin et al. (2017) [34]. They used the correlation of the equation between the volume loss with the hardness and specific energy of the materials to predict the abrasive conditions of the materials. From the hardness values, it is possible to infer that the nanoparticles are a hardening material by themselves, which confers a certain degree of stiffness–plasticity to the rubber composites.

Regarding the density of rubber nanocomposites (as reported in Table 4), carbon black as filler increased the CR/NR/BR rubber matrix by about 6%, while the organoclays did so by 2%. This means an advantage for organoclays as fillers, because the composites with low densities are easier to manage in industrial processes.

### 3.2. X-Ray Diffraction Analysis 

**Figure 1:**
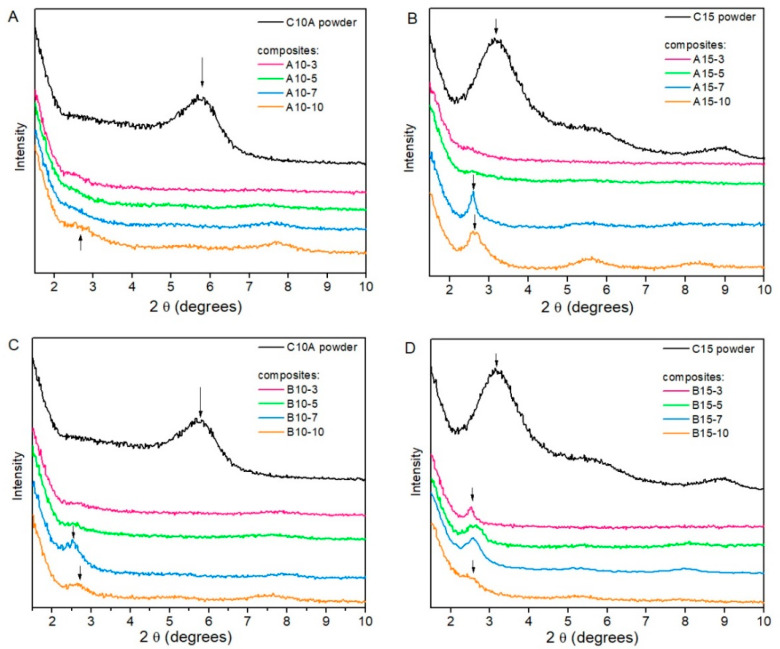
X-ray diffraction patterns of (**A**) Cloisite^®^ 10A powder (C10A) and rubber nanocomposites with C10A. (**B**) Rubber nanocomposites with CB and C10A. (**C**). Cloisite^®^ 15 powder (C15) and rubber nanocomposites with C15. (**D**). Rubber nanocomposites with CB and C15.

The X-ray diffraction patterns of nanocomposites and powdered organoclays can be seen in Figure 1. The pattern of C10A (shown in Figure 1A), displayed a peak at 2θ = 5.72° related to the basal plane family (001) of the organoclay. According to Bragg’s law, this was equivalent to an intergallery spacing (d_001_) of 19.41 Å. This d_001_ value is backed by Mohomane et al. (2011) [35]. Regarding composites using C10A as filler (as seen in Figure 1A), the patterns of A10-3, A10-5 and A10-7 did not show the peak related to the intergallery spacing of C10A. This could be because the C10A organoclay was exfoliated in these composites. Moreover, the pattern of the A10-10 composite showed that the peak related to d_001_ shifted at 2θ = 2.70° (d_001_ = 41.12 Å), which probably showed organoclay intercalation at high concentrations, such as 10 phr. The pattern of C15 (shown in Figure 1B), displayed its basal reflection characteristic peak at 2θ = 3.15° (d_001_ = 35.24 Å) [35,36,37]. It could be seen how the peak shifted in the patterns of A15-7 and A15-10, at 2θ = 2.60° (d_001_ = 42.70 Å) and 2θ = 2.62° (d_001_ = 42.37 Å), respectively. However, the same was not observed in the patterns of A15-3 and A15-5 (as seen in Figure 1B). From these results, in which organoclays were acting alone as fillers in the CR/NR/BR nanocomposites (Figure 1A,B), it was shown that C10A was better dispersed than C15 at 7 and 10 phr and equally at 3 and 5 phr, respectively. This could explain why the better mechanical properties in tension, particularly tensile strength, were obtained at 7 phr with C10A and at 5 phr with C15.

The patterns of composites with carbon black and C10A organoclays as fillers (in Figure 1C) showed that the organoclay could be exfoliated in composites at 3 phr and 5 phr, but at 7 phr and 10 phr it was intercalated. Thus, the patterns of the B10-3 and B10-5 composites did not show any peak related to the organoclay d_001_. While, the patterns of the B10-7 and B10-10 composites showed a peak at 2θ = 2.52° (d_001_ = 44.05 Å) and 2.68° (d_001_ = 41.42 Å), respectively. Composites with carbon black and C15 organoclay (shown in Figure 1D) displayed organoclay intercalation, because the peak related to its d_001_ was observed at lower angles. Thus, in the pattern of the B15-3 composite, the peak was shifted at 2θ = 2.52° (d_001_ = 44.05 Å), while for B15-5, B15-7 and B15-10 it was shifted at 2θ = 2.58° (d_001_ = 43.03 Å) in all of them.

### 3.3. Thermogravimetric Analysis

**Figure 2:**
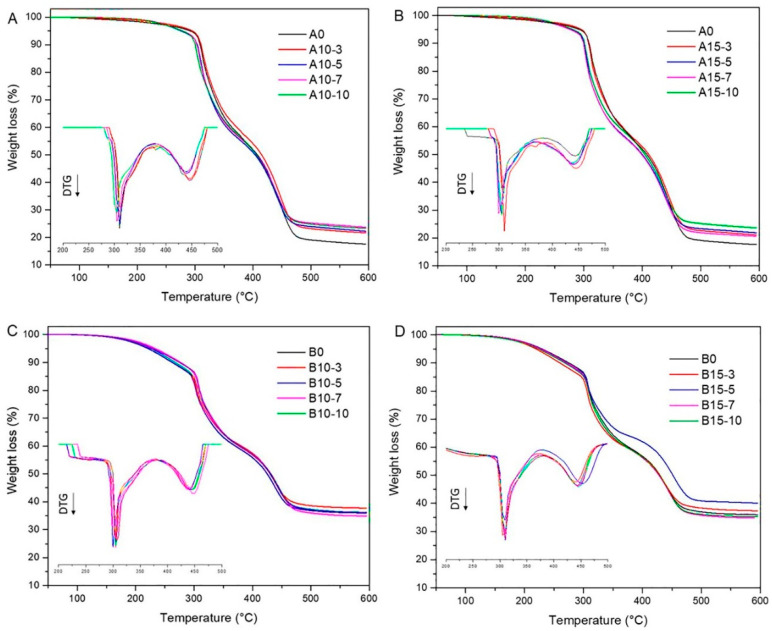
Thermogravimetric curves of the rubber nanocomposites containing different types and concentrations of fillers (**A**) Cloisite^®^ 10A. (**B**) Cloisite^®^ 15. (**C**,**D**) Cloisite^®^ 10 A and Cloisite^®^ 15 plus carbon black N330, respectively.

The rubber nanocomposites’ thermal stability and degradation temperature can be evaluated by thermogravimetric analysis (TGA) (Figure 2). All rubber nanocomposites exhibited similar thermal behavior regarding mass loss, with two decomposition steps displayed. The first step, between 50 and 300 °C with a weight loss of about 20.0%, was associated with low molecular weight substance losses. In this step, the degradation corresponds to the decomposition of volatiles and CR rubber, mainly due to dehydrochlorination. The rapid thermal degradation was explained by autocatalytic chloroprene chain pyrolysis, with the formation of HCl and chloroprene monomer, which was previously reported by Das et al. (2008) [36]. In the A0 composite, the degradation started at around 230 °C and reached a maximum rate at around 311 °C. It was observed that nanocomposites without CB and with a 7 and 10 phr filler concentration of organoclay C10A and C15 caused a decrease around 5 and 7 °C in the first decomposition step, respectively, while in the compounds with carbon black it was 2 and 3 °C. The octadecylamine hydrochloride ions weakly bound to the surface of the montmorillonite, that is, ions adsorbed on the hydroxyl groups at the edges of the clay sheets [30,31]. It is speculated that a higher concentration of filler generated a decrease in the dispersion and distribution of these inside the rubber matrix, producing agglomerates that facilitated the transfer of volatiles, thereby hastening matrix degradation, which in turn caused a decrease in the thermal stability of the composites [38].

A second pronounced loss of weight (about 55.0%) was presented in a range of 310–450 °C, and it corresponds to the release of tar, volatiles and decomposition of NR and BR rubbers. In this step, the nanocomposites (without carbon black) at a 7 and 10 phr filler concentration of C10A produced a decrease of around 2 °C, while for C15 organoclay it was 5 °C. The composite containing carbon black presented a decomposition temperature increase of about 4 °C. At higher temperatures above 600 °C, weight loss is associated with carbonaceous residues elimination, ash and mineral filler [39]. As expected, the A0 composite exhibited the lower char residue (18%), while the cured rubber composites with organoclay and CB showed higher char content (38%). Due to the inorganic layers of nanoclays and the carbonaceous material, composites were thermally stable and do not degrade much, even at 800 °C.

### 3.4. FT-IR Analysis

**Figure 3:**
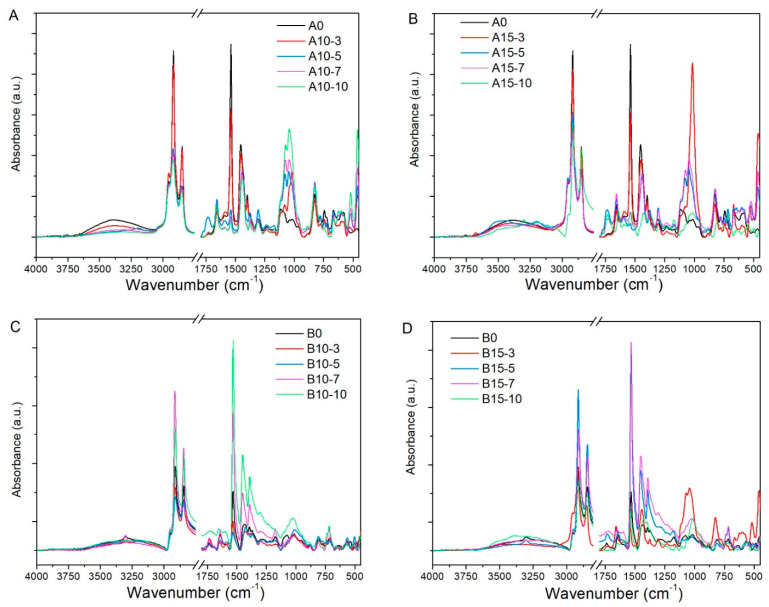
FT-IR spectra of rubber nanocomposites containing different types and amounts of fillers. (**A**) Cloisite^®^ 10A. (**B**) Cloisite^®^ 15. (**C**,**D**) Cloisite^®^ 10A and Cloisite^®^ 15 plus carbon black N330, respectively.

Figure 3 shows the FT-IR spectra of the CR/NR/BR matrix, nanocomposites with Cloisite^®^ 10A, Cloisite^®^ 15 and carbon black. For all spectra, a broad band of around 3380 cm^−1^ (νs –N–H) can be observed, which is due to the presence of proteins in NR [40]. The weak band that appeared at 2954 cm^−1^ (νas –CH_3_) the strong bands at 2914 cm^−1^ (νs –CH_3_) belong to natural rubber, and the vibration located at 2849 cm^-1^ (νs –CH_2_, νs –CH_3_) belongs to chloroprene [41].

On the other side, the small band at 1733 cm^−1^ (ν –(C=O)–O–) can be due to the lipid presence in NR, as previously reported [40,42]. Whereas, the known Amide I band at 1657 cm^−1^ (ν –(C=O)–NH) and the sharp Amide II band at 1538 cm^−1^ (β –N–H + ν –C–N) are specific of peptide bonds, confirming the protein presence [43]. The vibrational bands at 1453 cm^−1^ (δ –CH_2_ + ρ –CH_3_), 1399 cm^−1^ (δas –CH_3_), and 1305 cm^−1^ (δs –CH_3_) are characteristic of the carbon-hydrogen bonds of the macromolecules [42]. Other vibration bands associated to the rubber mixture are 1077 cm^−1^ (τ –CH_2_), 1045 cm^−1^ (ρ –CH_3_), 1015 cm^−1^ (ν C–C), 1036 cm^−1^ (ρ –CH_3_), 1014 cm^−1^ (ν C–C), 986 cm^−1^ (τ C=C) and 744 cm^−1^ (ρ –CH_2_) [40,42].

In the rubber matrices and rubber nanoclay reinforced samples, a small band is located at 1376 cm^−1^ (δ^as^ -CH_3_). This absorption appears as a shoulder in carbon black reinforced samples but shifted to lower wavelengths (1373 cm^−1^). This finding agrees with previously published results from other authors that observed a similar band absorption shifting in carbon black filling NR material [44]. These authors consider it as proof of the good physical chemical interaction between both materials, which explains the reinforcing character of the filler network in the rubber matrix.

Other vibrational bands associated with the rubber mixture are 1096 cm^−1^ (τ –CH_2_), 1039 cm^−1^ (ρ –CH_3_), 1014 cm^−1^ (ν C–C), 986 cm^−1^ (τ C=C) and 744 cm^−1^ (ρ –CH_2_) [40,42,44]. The band appearing at 824 cm^−1^ is due to the -CH_2_ rocking near the double bond, which is a strong band of primary importance in chloroprene, along with the band at 665 cm^−1^ (ν –C–Cl) [14].

New strong bands at 1077 cm^−1^ and 1045 cm^−1^ appeared in nanocomposites with nanoclays, but not in the non-reinforced material. The 1045 cm^−1^ (ρ –CH_3_) band could come from the organic modifiers of nanoclays [45]. Moreover, the stretching vibration of the Si-O bond that is typical of nanoclays is also observed near to this region at 1047 cm^−1^. On the other side, rubber nanocomposites reinforced with organoclays presented two absorption bands with maximums at 1077 and 1014 cm^−1^, and it was due to the intercalation of organoclays because of the rubber chains that came into the gallery of clays [45,46].

The absorption band that appears in all the composites from 455–462 cm^−1^ (ν S–S) could be due to the sulfide linkage [47], but it can also correspond to the bending of the Si–O bond present in the nanoclays. By incorporating the nanoclays into the rubber compounds, the intensity of this band increased as well, reaffirming the intercalation of the organoclays because of the rubber chains into its gallery [48]. It is a fact that during the vulcanization process, the –C=C– bonds are broken due to sulfur addition. Then sulfur atoms act as chain cross-linkers inside rubber to form mono, di and poly-sulfide linkages. As for the type of sulfur conventional systems used in the recipes of the nanocomposites of this work, it is expected that it was mainly due to the mono-sulfide linkages [24]. Finally, for carbon black-filled samples, the 1077 cm^−1^ band shifted to 1070 cm^−1^, and the 1042 cm^−1^ band is displayed as a medium intensity broad band at 1027 cm^−1^. These shifts could be related to the strong interaction of reinforcing materials inside the rubber matrix and potential interaction with sulfur bridges. Such physical chemical interactions could play a significant role in the mechanical properties of the resulting material.

### 3.5. Cure Characteristics

**Figure 4:**
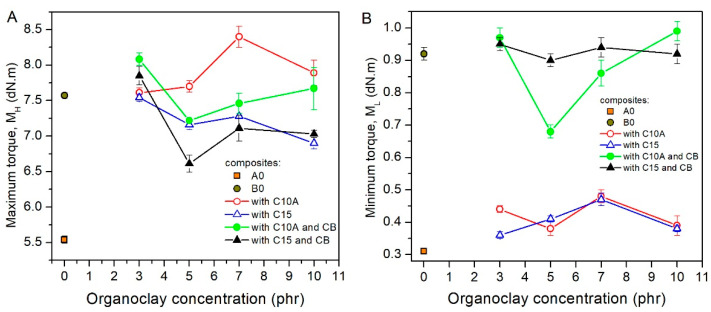
Curing torques of rubber nanocomposites. (**A**) Maximum torques of cured nanocomposites (M_H_). (**B**) Minimum torques of nanocomposites before the curing start (M_L_).

In the case of the maximum torque (M_H_) of the CR/NR/BR matrix, it was increased at 3 phr in C10A and C15 by 37% and 36%. However, at higher concentrations than 3 phr, the increase in M_H_ was not significant (see Figure 4A). It was evidenced that organoclays did not cause the inactivation of curing agent additives [36], and that the crosslinking of the cured composites was favored at low concentrations of the organoclay. While in composites with carbon black, the M_H_ increased by 36% in contrast to the A0 composite, which was even lower than that achieved by the organoclays. This result was consistent with modulus at 300% and the tensile strength of the CR/NR/BR matrix, in which organoclay could improve these properties at low concentrations (5 or 7 phr), even without carbon black.

Composites with the C10A and C15 organoclays at any concentration (blue and red lines) increased the M_L_ of the CR/NR/BR rubber matrix by more than 54%, in comparison to the A0 composite (orange scatter) (Figure 4B). However, this increase was not as high as that of the composite with only carbon black. For instance, in the B0 composite, the M_L_ value was higher by 190% than that of the A0 composite. In this sense, as M_L_ is associated with the viscosity of the raw rubber composites, the organoclays as fillers would not hinder its industrial conformation, for instance, through the calendering or extrusion processes.

**Figure 5:**
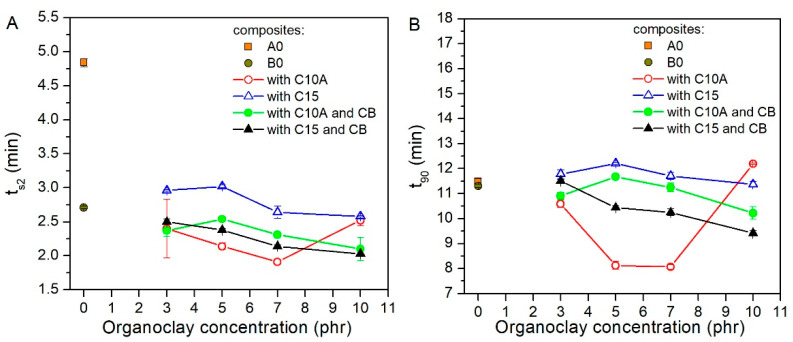
Curing times of rubber nanocomposites. (**A**) Vulcanization start time (t_s2_). (**B**) Time at 90% for vulcanization degree (t_90_).

Figure 5A showed that composites with organoclays at 3 phr decreased the t_s2_ of the CR/NR/BR matrix by a 50% to approximately 40%. This effect was similar to that caused by the carbon black. Composites B10-10 and B15-10 had a t_s2_ lower than B0 by 23% and 25%, respectively. Respecting the curing time (t_90_) (Figure 5B) of the CR/NR/BR matrix (A0), it was affected differently by organoclays. In this sense, the real effect was observed in composites with only C10A as filler at 5 and 7 phr and in composites with C15 and CB as fillers, also at 5 and 7 phr. In both cases, t_90_ was lower than this of A0 or even B0. The C10A organoclay lowered the t_90_ values, except when it was used at a 10 phr concentration, this being the last point out of trend. In contrast, the C15 organoclay slightly raised t_90_ values or kept them almost constant. In another work, it was indicated that such a remarkable decrease in t_90_ values means that the curing reaction of the rubber matrix is being favored, which could be because of the action of a functional group in organoclay and the basic nature of the CB [25].

## 4. Conclusions

The CR/NR/BR nanocomposites that were prepared and characterized in this work showed that C10A and C15 organoclays used as fillers have some advantages over the carbon black N330. Thus, the improved physical mechanical properties of these nanocomposites are achieved at very low organoclay concentrations (less than 7 phr). Moreover, these nanocomposites with organoclays could equal or even exceed that which is obtained with CB at concentrations as high as 40 phr. Moreover, the use of a low concentration of a filler is essential to avoid high viscosity in the rubber crude mix of nanocomposites and low densities; in the opposite case, their conformation process would be difficult.

All the results evidenced that the interaction between organoclays and carbon black was not synergistic. Although this was because of the high content of CB, there probably exists an optimal ratio between the C10A or C15 organoclays with N330 carbon black, in which the synergism would be evident. The main disadvantage of organoclays is their high cost, but this could be balanced by the low content necessary to obtain novel nanocomposites with excellent performance.

The FTIR-ATR analysis was an important technique to prove the reinforcing interaction of nanoclays in rubber nanocomposites based on the evidence of the intercalation phenomenon found. Carbon black also exhibited displaced bands characteristic of its interaction with rubber matrices.

The compounding method was also important for helping with the interaction of organoclay and the rubber phases. Thus, mixing organoclays directly with the raw chloroprene in the early stages of compounding is recommended.

## 5. Patents

Chilean Patent Application No. 202003082, 2020.

## Figures and Tables

**Table 1:** Characteristics of nanofillers studied in this work (information retrieved from the supplier).

Filler	Intergallery Spacing d(001) (Å)	Organic Modifier	Particle Size (nm)
Organoclay Cloisite 10 A (C10A)	19.0	Dimethyl, benzyl, hydrogenated tallow, quaternary ammonium chloride.	-
Organoclay Cloisite 15 (C15)	36.3	Dimethyl, dehydrogenated tallow, quaternary ammonium chloride.	-
Carbon black N330 (CB)	-	-	29

**Table 2:** Recipes for rubber nanocomposite. Quantities are given in parts per hundred rubber (phr).

Composite Code	Chloroprene Rubber (CR)	Natural Rubber (NR)	Butadiene Rubber (BR)	Cloisite^®^ 10 A (C10A)	Cloisite^®^ 15 (C15)	Carbon Black (CB)	Zinc Oxide (ZnO)	S	Others
A0	69.6	25.3	5.1	0.0	0.0	0.0	2.8	2.2	6.8
A10-3	69.6	25.3	5.1	3.0	0.0	0.0	2.8	2.2	6.8
A10-5	69.6	25.3	5.1	5.0	0.0	0.0	2.8	2.2	6.8
A10-7	69.6	25.3	5.1	7.0	0.0	0.0	2.8	2.2	6.8
A10-10	69.6	25.3	5.1	10.0	0.0	0.0	2.8	2.2	6.8
A15-3	69.6	25.3	5.1	0.0	3.0	0.0	2.8	2.2	6.8
A15-5	69.6	25.3	5.1	0.0	5.0	0.0	2.8	2.2	6.8
A15-7	69.6	25.3	5.1	0.0	7.0	0.0	2.8	2.2	6.8
A15-10	69.6	25.3	5.1	0.0	10.0	0.0	2.8	2.2	6.8
B0	69.6	25.3	5.1	0.0	0.0	40.0 *	2.8	2.2	6.8
B10-3	69.6	25.3	5.1	3.0	0.0	40.0 *	2.8	2.2	6.8
B10-5	69.6	25.3	5.1	5.0	0.0	40.0 *	2.8	2.2	6.8
B10-7	69.6	25.3	5.1	7.0	0.0	40.0 *	2.8	2.2	6.8
B10-10	69.6	25.3	5.1	10.0	0.0	40.0 *	2.8	2.2	6.8
B15-3	69.6	25.3	5.1	0.0	3.0	40.0 *	2.8	2.2	6.8
B15-5	69.6	25.3	5.1	0.0	5.0	40.0 *	2.8	2.2	6.8
B15-7	69.6	25.3	5.1	0.0	7.0	40.0 *	2.8	2.2	6.8
B15-10	69.6	25.3	5.1	0.0	10.0	40.0 *	2.8	2.2	6.8

* This carbon black (CB) concentration was selected because it is an approximate value and has been frequently used in the industry to reinforce chloroprene rubbers.

**Table 3:** Mechanical properties of the rubber nanocomposites. The data presented are the average values and their standard deviation.

Composite Code	Tensile Strength (MPa)	Elongation at Break (%)	M 300 (MPa)	Tear Strength (N mm^−1^)
A0	5.36 ± 1.52	730.91 ± 95.97	1.35 ± 0.03	16.29 ± 1.56
A10-3	6.85 ± 0.80	739.10 ± 41.61	1.74 ± 0.04	21.08 ± 0.80
A10-5	11.85 ± 1.09	832.15 ± 29.95	2.41 ± 0.36	24.05 ± 1.20
A10-7	13.65 ± 0.76	836.82 ± 11.38	3.05 ± 0.05	31.51 ± 2.29
A10-10	10.14 ± 0.79	768.33 ± 23.77	2.88 ± 0.07	30.76 ± 1.91
A15-3	9.95 ± 1.13	840.35 ± 37.73	1.88 ± 0.03	19.09 ± 0.87
A15-5	13.42 ± 0.83	933.19 ± 45.89	2.24 ± 0.20	25.13 ± 1.45
A15-7	12.40 ± 0.85	915.79 ± 13.82	2.31 ± 0.13	28.82 ± 2.84
A15-10	10.01 ± 0.90	797.01 ± 41.74	2.70 ± 0.07	26.58 ± 2.32
B0	9.95 ± 0.84	477.64 ± 15.41	6.71 ± 0.18	27.97 ± 0.66
B10-3	8.55 ± 0.82	338.55 ± 22.87	7.49 ± 0.15	26.64 ± 2.49
B10-5	7.44 ± 1.01	519.05 ± 6.81	6.54 ± 0.13	27.42 ± 2.68
B10-7	10.73 ± 0.72	399.15 ± 16.38	7.74 ± 0.35	29.83 ± 0.46
B10-10	8.83 ± 1.10	370.92 ± 31.69	6.86 ± 0.18	28.73 ± 2.82
B15-3	12.77 ± 0.71	466.23 ± 25.73	7.19 ± 0.22	26.87 ± 2.53
B15-5	7.02 ± 0.63	455.75 ± 17.58	6.35 ± 0.09	29.38 ± 2.14
B15-7	12.43 ± 0.40	470.10 ± 18.88	6.97 ± 0.14	30.85 ± 2.23
B15-10	7.31 ± 0.02	310.98 ± 1.47	7.11 ± 0.10	33.06 ± 2.56

**Table 4:** Physical properties of rubber nanocomposites and loss of material by abrasion.

Composite Code	Hardness (Shore A)	Loss of Volume by Abrasion (mm^3^)	Reduction of Volume Loss (%)	Density (g/cm^3^)
A0	42 ± 0	449.97 ± 5.88	-	1.1356 ± 0.0011
A10-3	47 ± 0	251.29 ± 13.09	44.15	1.1479 ± 0.0026
A10-5	51 ± 0	243.42 ± 9.81	45.96	1.1497 ± 0.0022
A10-7	56 ± 1	251.35 ± 7.13	44.14	1.1460 ± 0.0051
A10-10	54 ± 1	269.68 ± 1.37	40.06	1.1607 ± 0.0042
A15-3	50 ± 1	246.23 ± 5.05	45.28	1.1444 ± 0.0010
A15-5	54 ± 1	241.73 ± 7.75	46.29	1.1479 ± 0.0022
A15-7	62 ± 1	251.99 ± 15.73	43.99	1.1518 ± 0.0016
A15-10	53 ± 0	399.11 ± 26.92	11.3	1.1587 ± 0.0004
B0	59 ± 1	288.86 ± 6.69	-	1.2016 ± 0.0007
B10-3	61 ± 1	242.07 ± 2.62	16.2	1.2101 ± 0.0016
B10-5	64 ± 1	243.56 ± 11.15	15.68	1.2121 ± 0.0064
B10-7	61 ± 1	240.51 ± 8.38	16.74	1.2164 ± 0.0015
B10-10	63 ± 0	266.96 ± 3.72	7.51	1.2197 ± 0.0010
B15-3	63 ± 1	227.12 ± 2.30	21.37	1.2040 ± 0.0013
B15-5	66 ± 1	247.14 ± 12.32	14.44	1.2088 ± 0.0030
B15-7	63 ± 0	216.48 ± 3.35	25.06	1.2186 ± 0.0004
B15-10	64 ± 0	249.51 ± 3.86	13.62	1.2124 ± 0.0014
